# Psychosocial and Economic Burden on Families of Children With Cerebral Palsy: A Correlation With Locomotor Severity

**DOI:** 10.7759/cureus.76794

**Published:** 2025-01-02

**Authors:** Falak Naaz, Bibhu Prasad Nayak, Sumanta Panigrahi, Nirmal Kumar Mohakud

**Affiliations:** 1 Medicine, Sri Jagannath Medical College and Hospital, Puri, IND; 2 Pediatrics, Dharanidhar Medical College and Hospital, Kendujhar, IND; 3 Pediatrics, Sriram Chandra Bhanja Medical College and Hospital, Cuttack, IND; 4 Pediatrics, Kalinga Institute of Medical Sciences (KIMS) Kalinga Institute of Industrial Technology (KIIT), Bhubaneswar, IND

**Keywords:** cerebral palsy, economic burden, gmfcs, psychosocial stress, rehabilitation

## Abstract

Background: Cerebral palsy (CP) is a condition that often has significant psychosocial and economic impacts on the caregivers of affected children.

Objective: This study aimed to assess the association between the Gross Motor Function Classification System (GMFCS) level and the psychosocial and economic impact on caregivers of children with CP.

Methodology: A hospital-based cross-sectional observational study was conducted on children with CP aged 2-14 years, admitted to the Inpatient Department (IPD) or attending the District Early Intervention Center (DEIC) for physiotherapy at a teaching hospital in Odisha, from December 2020 to November 2022. In DEIC, appropriate screening and therapy as per requirement is given to the high-risk infants. Early detection of CP is done. Children with CP come here with their parents for physiotherapy, occupational therapy, hearing, vision, and development assessment. Tools used included the GMFCS - Expanded and Revised (GMFCS-ER), a five-level classification system, the Modified Updated Kuppuswamy Socioeconomic Scale (2021) for socioeconomic status (SES), and the Pai and Kapur Family Burden Interview Scale.

Results: A total of 160 children with CP were included in the study, with 98 males and 62 females, resulting in a male-to-female ratio of 1.58:1. Out of 160 children with CP, the socioeconomic distribution showed that 73 (45.6%) belonged to the upper-lower class, 68 (42.5%) to the lower-middle class, 9 (5.6%) to the lower class, and 10 (6.3%) to the upper-middle class. Regarding functional levels of 160 children with CP, 22 (13.8%) of children were in GMFCS class I, 30 (18.8%) in class II, 16 (10%) in class III, 17 (10.6%) in class IV, and 75 (46.7%) in class V. Financially, out of 160 families of children with CP, 75 (46.9%) families were moderately burdened, 84 (52.5%) were severely burdened, and only 1 (0.6%) reported no financial burden. Regarding psychosocial impact, 94 (58.8%) families experienced moderate disruption of family leisure, while 44 (27.5%) experienced severe disruption. Physical health was moderately affected in 73 (45.6%) families, and 14 (8.8%) reported a severe impact. Mental health was moderately affected in 88 (55%)of families, while 33 (20.6%) experienced severe mental health issues. There was a statistically significant association between the GMFCS level of the child and the psychosocial and economic burden on families.

Conclusions: The study concludes that higher GMFCS levels in children with CP are associated with a greater psychosocial and economic burden on their families.

## Introduction

Cerebral palsy (CP) is recognized as the most prevalent childhood disability, affecting approximately 2 to 3 children per 1000 live births globally [1,2,3]. In India, the prevalence is slightly higher, at 2.95 per 1000 surveyed children [4]. CP encompasses a group of permanent movement disorders that result from damage to the developing brain, either during pregnancy, childbirth, or shortly after birth. These motor impairments affect the child's ability to control their muscles and body movements, often leading to lifelong challenges in mobility, posture, and coordination [5]. The disorder is associated with various degrees of severity, classified through the Gross Motor Function Classification System (GMFCS), which ranges from level I (mild impairment) to level V (severe impairment) [6].

The care and management of children with CP rely heavily on a multidisciplinary team approach, involving pediatricians, neurologists, physiotherapists, occupational therapists, and educators [7]. However, the central role in the daily care and rehabilitation of these children is often played by parents and other family members [8]. These caregivers must be actively involved in every stage of the child's treatment, including managing healthcare appointments and physiotherapy sessions and ensuring adherence to treatment protocols. While this involvement is crucial for the child's well-being, it places a significant psychosocial and economic burden on the family [9].

The psychosocial impact on caregivers includes emotional stress, mental health challenges, disruption of social life, and a feeling of isolation, as families may struggle to balance the needs of their child with CP and the demands of their personal and professional lives. Financially, the continuous need for medical treatments, rehabilitation, assistive devices, and specialized care can lead to substantial economic strain, particularly for families with lower socioeconomic status (SES). Additionally, the time and energy required for caregiving may limit the ability of one or both parents to engage in full-time employment, further exacerbating financial difficulties [10].

This study seeks to quantify the psychosocial and economic stress experienced by families of children with CP and identify key factors associated with these challenges. By correlating the child's functional ability, as assessed by the GMFCS, with the family's SES and the burden they experience, the study aims to provide valuable insights into the predictors of caregiver stress. Understanding these relationships can help healthcare professionals design more supportive interventions for families, improving both the quality of life for children with CP and their caregivers.

## Materials and methods

This hospital-based cross-sectional observational study was conducted at Sriram Chandra Bhanja Medical College and Hospital (SCBMCH) and Sardar Vallabhbhai Patel Post Graduate Institute of Pediatrics (SVPPGIP), Cuttack, Odisha, from December 2020 to November 2022. The study population comprised children aged 2 to 14 years diagnosed with CP. Taking CP prevalence as 2.95 children and confidence interval as 95%, our sample size was calculated as 336. As the study was done during the COVID era from December 2020 to December 2022, fewer children with CP attended our District Early Intervention Center (DEIC) for physiotherapy. A total of 160 children with CP from the Inpatient Department (IPD) and DEIC, who met the inclusion criteria, were included in the study. In the DEIC, children with physical challenges, including CP, autism, attention-deficit/hyperactivity disorder (ADHD), and other conditions, come to receive various therapies. This is a government setup available in all districts of India. It is located at the district headquarters hospital. Multidisciplinary departments such as physiotherapy, occupational therapy, psychology, ophthalmology, dentistry, and audiology are all present. Here, early intervention is done for high-risk infants to whom the pediatrician refers. Children with CP come here with their parents for physiotherapy, occupational therapy, hearing, vision, and development assessment. Parental consent was obtained before their participation. Children aged 2-14 years who were diagnosed with CP were included. Children with associated progressive neurological, metabolic, neuromuscular, neurodegenerative, rheumatological, or other chronic debilitating disorders, incomplete questionnaires, and critically ill were excluded. Tools used are the Gross Motor Function Classification System - Expanded and Revised (GMFCS-ER) [11], the Modified Updated Kuppuswamy Socioeconomic Scale 2021 [12], and the Pai and Kapur Family Burden Interview Scale (FBIS), as represented in the Appendix [10,13].

GMFCS-ER is a five-level classification system designed to assess gross motor function in children with CP. First introduced in 1997 and revised in 2008, the GMFCS-ER evaluates children's ability to perform motor activities such as sitting, walking, and mobility. Each child was classified into one of the five GMFCS levels for this study. A family's SES is based on three parameters: the head of the family's education level, occupation, and overall family income. The scale classifies families into five socioeconomic classes: upper, upper-middle, lower-middle, upper-lower, and lower, with scores ranging from 3 to 29. The FBIS is a semi-structured interview schedule comprising 24 items grouped into six dimensions: financial burden, disruption of routine family activities, disruption of family leisure, disruption of family interactions, effect on the health of others, and effect on the mental health of others. Responses are rated on a three-point Likert scale (no burden, moderate burden, severe burden). The reliability and validity of the scale were previously reported to be above 0.78, establishing it as a reliable tool for assessing family burden. In this study, the interview schedule was translated into Odia and Hindi for ease of use.

Data were collected through structured interviews with parents of children with CP attending the IPDs of SCBMCH, SVPPGIP, and DEIC at SVPPGIP. Data were collected by the researcher who was doing post-graduation in pediatrics. The pediatrics specialists posted in DEIC were also trained to collect the data in pre-designed Performa. The uniformity was done by taking the data of previously diagnosed cases of CP who had come to DEIC for physiotherapy or were admitted to IPD for any complication. Data were collected using a pre-designed proforma, and each child’s motor disability was categorized using the GMFCS. The burden on families was assessed through interviews using the Pai and Kapur FBIS, translated into regional languages. All responses were recorded and entered into an MS Excel spreadsheet, and variables were coded accordingly.

Data were analyzed using SPSS version 25 software (IBM Corp., Armonk, NY). Categorical data were expressed in percentages, and associations between two categorical variables were assessed using the chi-square test. A *P*-value of less than 0.05 was considered statistically significant. Ethical approval was obtained from the Ethical Committee of SCBMCH, Cuttack. Informed consent was secured from all participants' parents in both English and the regional Odia language.

## Results

A total of 160 cases of CP were included in the study. Of these, 98 (61.3%) were males and 62 (38.8%) were females. Most of the children were aged between 6 and 14 years, with a mean age of 7 ± 3.17 years. SES, as determined by the Modified Updated Kuppuswamy Socioeconomic Scale 2021, revealed that out of 160 families, 9 (5.6%) belonged to the lower class, 73 (45.6%) to the upper-lower class, 68 (42.5%) to the lower-middle class, and 10 (6.3%) to the upper-middle class. Based on the GMFCS-ER classification, the distribution of 160 children with CP was as follows: 22 (13.8%) in level I, 30 (18.8%) in level II, 16 (10%) in level III, 17 (10.6%) in Level IV, and 75 (46.9%) in Level V. The mean age of fathers was 37.1 ± 5.97 years, while the mean age of mothers was 31.78 ± 5.45 years. In terms of CP types, 83 children (52%) had spastic quadriplegia, with all four limbs equally affected by hypertonia; 31 children (19.3%) had diplegia, with hypertonia predominantly affecting the lower limbs more than the upper limbs; 32 children (20%) had hemiplegic CP, primarily affecting the right upper and lower limbs; 12 children (8%) had a mixed type of CP, presenting as spastic-dyskinetic; and 2 children (1%) had the hypotonic variety. Mostly spastic quadriplegia and diplegia children belonged to GMFCS levels IV and V.

The majority of families reported the disruption of routine family activities. Specifically, 13 (8.1%) of families experienced no disruption, 106 (66.3%) reported moderate disruption, and 41 (25.6%) indicated severe disruption. The primary reason for the disruption of routine family life was the frequent need for hospital visits. Additionally, caring for the dependent child, such as feeding, bathing, dressing, managing the child not attending school, or the inability to assist with household chores, also contributed significantly to the disruption. This results in negligence of other family members, especially siblings. The GMFCS groups IV and V experienced more disruption. In terms of family leisure, 22 (13.8%) families experienced no disruption, while 94 (58.8%) faced moderate disruption and 44 (27.5%) reported severe disruption. Many families noted that their usual recreational activities had ceased, family holidays were affected, and other leisure activities had been postponed due to their child’s illness (Table 1).

**Table 1:** Frequency distribution of severity of financial burden and disruption in routine family activities and leisure among children 2 to 14 years.

Type of financial burden (*n *= 160)	No burden	Moderate burden	Severe burden
A. Financial burden as a whole	1 (0.6%)	75 (46.9%)	84 (52.5%)
A1. Loss of patient’s income and its effect on family income	153 (95.6%)	7 (4.4%)	0 (0.0%)
A2. Loss of income of any other member due to patient	36 (22.5%)	65 (40.6%)	59 (36.9%)
A3. Expenditure incurred due to patients and treatment and its effect on family finances	3 (1.9%)	61 (38.1%)	96 (60.0%)
A4. Expenditure incurred due to extra arrangements	12 (7.5%)	81 (50.6%)	67 (41.9%)
A5. Loans taken, its effect on family finances and savings spent	58 (36.3%)	49 (30.6%)	53 (33.1%)
A6. Any other planned activity was put off because of financial pressure owing to the patient’s illness	26 (16.3%)	81 (50.6%)	53 (33.1%)
Frequency distribution of Disruption of Routine Family Activities			
B. Disruption of routine family activities	13 (8.1%)	106 (66.3%)	41 (25.6%)
B7. The patient not going to school, college, work, etc.	65 (40.6%)	34 (21.3%)	61 (38.1%)
B8. The patient not helping with the household work	55 (34.4%)	48 (30.0%)	57 (35.6%)
B9. Disruption of activities of other family members	45 (28.7%)	60 (38.2%)	52 (33.1%)
B10. The patient’s behavior disrupting activities	65 (40.6%)	60 (37.5%)	35 (21.9%)
B11. Neglect of the rest of the family due to the patient’s illness	58 (36.3%)	45 (28.1%)	57 (35.6%)
Frequency distribution of disruption of family leisure			
C. Disruption of family leisure	22 (13.8%)	94 (58.8%)	44 (27.5%)
C12. Stopping normal recreational activities	32 (20.0%)	73 (45.6%)	55 (34.4%)
C13. The patient’s illness using up another person’s holiday/leisure time	34 (21.3%)	70 (43.8%)	56 (35.0%)
C14. The patient’s lack of attention to other members children and its effect on him/her	128 (80.0%)	29 (18.1%)	3 (1.9%)
C15. Any other leisure activity had to be abandoned due to the patient’s illness	34 (21.3%)	95 (59.4%)	31 (19.4%)

Family interaction was not disrupted in 48 (30%) of cases, moderately disrupted in 77 (48.1%) of cases, and severely disrupted in 35 (21.9%) of cases. The effect on the physical health of caregivers was reported as follows: 73 (45.6%) experienced no ill effects, 73 (45.6%) were moderately affected, and 14 (8.8%) were severely affected. Regarding the mental health of family members, 33 (20.6%) reported severe mental health effects, 88 (55%) experienced moderate effects, and 39 (24.4%) reported no impact on their mental health. Caregivers, particularly parents, often reported sleep disturbances, feelings of depression, and irritability as a result of caring for their child (Table 2).

**Table 2:** Frequency of distribution of severity of family interaction and effect on physical health among children 2 to 14 years.

Frequency distribution of disruption of family interaction			
Assessment question	No burden	Moderate burden	Severe burden
D. Disruption of family interaction	48 (30.0%)	77 (48.1%)	35 (21.9%)
D16. Any ill effect on the general atmosphere in the house	56 (35.0%)	46 (28.7%)	58 (36.3%)
D17. Do other members get into an argument	82 (51.2%)	44 (27.5%)	34 (21.3%)
D18. Have relatives and neighbors stopped visiting the family	128 (80.0%)	23 (14.4%)	9 (5.6%)
D19. Has the patient’s acute and chronic illness had any effect on the relationship	112 (70.0%)	27 (16.9%)	21 (13.1%)
D20. Has the family become secluded	124 (77.5%)	15 (9.4%)	21 (13.1%)
Effect on the physical and mental health of others			
E. Effects on the physical health of others	73 (45.6%)	73 (45.6%)	14 (8.8%)
E21. Have any other members suffered physical ill health	80 (50.0%)	63 (39.4%)	17 (10.6%)
E22. Has there been any other adverse effects on health	89 (55.6%)	54 (33.8%)	17 (10.6%)
F. Effects on the mental health of others	39 (24.4%)	88 (55.0%)	33 (20.6%)
F23. Have any other family members sought help for psychological illness	118 (73.8%)	27 (16.9%)	15 (9.4%)
F24. Have any other family members lost sleep, depressed, or irritable	17 (10.6%)	85 (53.1%)	58 (36.3%)

A significant association was found between the overall burden faced by families and the GMFCS level of the child (*P* < 0.001). As the GMFCS level increased, so did the severity of financial burdens, disruptions in routine family activities, family leisure, family interaction, and the physical and mental health of family members. These findings indicate that families with children with higher GMFCS levels face greater psychosocial and economic burdens. Detailed results are presented in Tables 3-4.

**Table 3:** Association of overall burden, economic burden, disruption of routine family activities, and leisure with GMFCS level. GMFCS, Gross Motor Function Classification System

Type of burden, family activities, and leisure	GMFCS level	Mildly severe	Moderately severe	Very severe	*P*-value
Overall burden	Level I	13 (59.1%)	0 (0.0%)	9 (40.9%)	<0.001
	Level II	7 (23.3%)	10 (33.3%)	13 (43.3%)	
	Level III	0 (0.0%)	7 (43.8%)	9 (56.3%)	
	Level IV	0 (0.0%)	0 (0.0%)	17 (100.0%)	
	Level V	0 (0.0%)	11 (14.7%)	64 (85.3%)	
Economic burden	Level I	1 (4.5%)	21 (95.5%)	0 (0.0%)	<0.001
	Level II	0 (0.0%)	22 (73.3%)	8 (26.7%)	
	Level III	0 (0.0%)	13 (81.3%)	3 (18.8%)	
	Level IV	0 (0.0%)	8 (47.1%)	9 (52.9%)	
	Level V	0 (0.0%)	11 (14.7%)	64 (85.3%)	
Disruption of routine family activities	Level I	6 (27.3%)	16 (72.7%)	0 (0.0%)	<0.001
	Level II	7 (23.3%)	20 (66.7%)	3 (10.0%)	
	Level III	0 (0.0%)	12 (75.0%)	4 (25.0%)	
	Level IV	0 (0.0%)	6 (35.3%)	11 (64.7%)	
	Level V	0 (0.0%)	52 (69.3%)	23 (30.7%)	
Disruption of family leisure	Level I	9 (40.9%)	13 (59.1%)	0 (0.0%)	<0.001
	Level II	7 (23.3%)	23 (76.7%)	0 (0.0%)	
	Level III	0 (0.0%)	13 (81.3%)	3 (18.8%)	
	Level IV	0 (0.0%)	12 (70.6%)	5 (29.4%)	
	Level V	6 (8.0%)	33 (44.0%)	36 (48.0%)	

**Table 4:** Association of disruption in family interaction and effects on the physical and mental health of others with GMFCS level. GMFCS, Gross Motor Function Classification System

Disruption in family	GMFCS level	Mildly severe	Moderately severe	Very severe	*P*-value
Disruption of family interaction	Level I	16 (72.7%)	6 (27.3%)	0 (0.0%)	<0.001
	Level II	10 (33.3%)	20 (66.7%)	0 (0.0%)	
	Level III	7 (43.8%)	6 (37.5%)	3 (18.8%)	
	Level IV	6 (35.3%)	8 (47.1%)	3 (17.6%)	
	Level V	9 (12.0%)	37 (49.3%)	29 (38.7%)	
Effects on the physical health of others	Level I	22 (100.0%)	0 (0.0%)	0 (0.0%)	<0.001
	Level II	17 (56.7%)	13 (43.3%)	0 (0.0%)	
	Level III	12 (75.0%)	4 (25.0%)	0 (0.0%)	
	Level IV	0 (0.0%)	17 (100.0%)	0 (0.0%)	
	Level V	22 (29.3%)	39 (52.0%)	14 (18.7%)	
Effects on the mental health of others	Level I	8 (36.4%)	14 (63.6%)	0 (0.0%)	<0.001
	Level II	13 (43.3%)	14 (46.7%)	3 (10.0%)	
	Level III	3 (18.8%)	13 (81.3%)	0 (0.0%)	
	Level IV	3 (17.6%)	7 (41.2%)	7 (41.2%)	
	Level V	12 (16.0%)	40 (53.3%)	23 (30.7%)	

## Discussion

The significance of this study lies in its comprehensive analysis of the psychosocial and economic impact on families of children with CP, specifically on the child's locomotor ability as classified by the GMFCS. Understanding these associations is crucial for clinicians and healthcare systems in designing effective family support strategies. CP is the most common childhood disability, and its impact on caregivers is profound, affecting multiple dimensions of life, including financial stability, family interactions, and physical and mental health. This study aims to provide detailed insights into the burden experienced by families and the correlation between the severity of the child's motor impairment and the magnitude of the burden. The major finding was the loss of income of the parents due to absence from work during working days. Adverse effects on the physical and mental health of parents in handling the dependent child. By identifying these associations, this study highlights the need for targeted interventions and holistic care models that address the medical and psychosocial needs of families dealing with CP. It emphasizes educating parents to visit DEIC at the appropriate times for early stimulation therapy for their child by physiotherapists, occupational therapists, and others, as well as the early recognition of hearing and vision loss with appropriate intervention therapy. Another targeted intervention by psychologists and physiotherapists is to recognize their family members or parents' mental and physical health illnesses and also treat them appropriately.

In the present study of 160 children with CP, males outnumbered females, with 61.3% of the children being boys and a male-to-female ratio of 1.58:1. A study by Najar et al. in Srinagar reported a male preponderance in their cohorts of children with CP [14]. The motor dysfunction, as classified by the GMFCS, showed that most children (46.9%) were in level V, the most severe category. Comparatively, a study by Dobhal et al. reported a higher distribution in level V [15]. In terms of CP types, the majority of children (52%) had spastic quadriplegia, which aligns with the findings from a study by Singhi et al. that report spastic CP as the most common form [16]. Our study also found similar results, with parents of children in GMFCS levels IV and V experiencing more difficulty and suffering.

The financial burden on caregivers was significant, with 52.5% of participants reporting severe financial strain and 46.9% experiencing moderate strain. Only 0.6% reported no financial burden. This financial hardship was mainly due to medical expenses, frequent hospital visits, and loss of income from work absences. Similar findings were reported by Laskar et al., where 69% of parents experienced severe financial difficulties, some even selling assets to cover their child’s medical expenses [17,18]. Our study showed a strong association between the GMFCS class and the severity of financial burden, indicating that families of children with more severe motor impairments are more likely to face significant financial challenges (*P *< 0.001). The government of Odisha with the help of the Indian government has a scheme called Rashtriya Bal Swasthya Karyakram (RBSK) where the paramedical teams visit rural areas to identify children with CP and bring them in government vehicles to the nearest DEIC for physiotherapy and drop back to their respective house. This helps the parents to save on the extra cost of transport.

Regarding the disruption of family activities, 66.3% of caregivers were moderately affected, while 25% experienced severe disruption. This disruption included difficulties managing daily household tasks, neglect of other family members, and decreased recreation or personal care time. Similar results were reported by Laskar et al., who found that nearly half of the families experienced moderate disruption [17]. Our study also found that the disruption of family leisure activities, such as attending social gatherings or engaging in recreational outings, was significantly associated with the GMFCS level (*P *< 0.001), with higher GMFCS levels correlating with more severe disruptions. 

Physical and mental health effects on caregivers were also notable [19,20]. While 45.6% of caregivers experienced no physical health issues, 45.6% reported moderate effects, and 8.8% reported severe physical health problems. Caregivers of children with higher GMFCS levels were more likely to report physical health issues (*P *< 0.001). Additionally, 55% of caregivers reported moderate effects on their mental health, while 20.6% experienced severe mental health challenges, such as depression, irritability, and sleep disturbances. Gignac reported similar findings, noting that economic constraints often prevented caregivers from seeking mental health support, despite high levels of psychological distress [21]. The overall psychosocial and economic burden faced by families was significantly correlated with the GMFCS level of the child, reinforcing the need for comprehensive support systems for families dealing with severe CP.

So our recommendation is parents should be aware of government programs like RBSK and other facilities related to disability from time to time. Parents and families should be aware of that to take advantage. The parents and families should also consult physicians and mental health professionals from time to time for their well-being. A robust information education communication (IEC) program requires the awareness of the parents to take advantage of government welfare schemes to relieve their stress. 

Limitations

This study is a single-center, hospital-based study. A multicenter study with a larger number of cases from different geographic locations or a community-based study would provide a better representation of a study population and additional insight into the topic. Not only the degree of a child's disability, but several other factors may be affecting caregiver stress. A more comprehensive study is required for a better understanding of the same.

## Conclusions

The results of this study demonstrate a clear and significant association between the degree of a child’s motor impairment, as classified by GMFCS, and the psychosocial and economic burden on their families. Higher GMFCS levels were correlated with increased disruption in family routines, greater financial strain, and more severe physical and mental health challenges for caregivers. These findings underscore the importance of tailored support systems for families, addressing both the medical needs of children with CP and the broad range of psychosocial and economic challenges faced by their caregivers. So the different government schemes regarding children with CP must be conveyed to each parent to take the benefit and relieve their stress. The system should include proper referral of children with CP to higher centers for their medical emergencies. Additionally, the RBSK team should screen family members to identify early signs of mental or physical illness and refer them to the district headquarters hospital or medical college for better treatment.

## Appendices

Appendix 

**Table 5:** Interview of the relatives on guidelines and note the ratings of each general category as well as each individual item in three point category Family Burden Interview Schedule (Pai and Kapur) [10]

A. Financial burden	No Burden Score-0	Moderate Burden Score-1	Severe Burden Score-2
A1. Loss of patient’s income and its effect on family income			
A2. Loss of income of any other member due to patient			
A3. Expenditure incurred due to patients and treatment and its effect on family finances			
A4. Expenditure incurred due to extra arrangements			
A5. Loans taken, its effect on family finances and savings spent			
A6. Any other planned activity put off because of financial pressure owing to patient’s illness			
Frequency distribution of Disruption of Routine Family Activities			
B. Disruption of routine family activities			
B7. Patient not going to school, college, work etc.			
B8. Patient not helping household work			
B9. Disruption of activities of other family members			
B10. Patient’s behaviour disrupting activities			
B11. Neglect of rest of the family due to patients’ illness			
Frequency distribution of Disruption of Family Leisure			
C. Disruption of family leisure			
C12. Stopping of normal recreational activities			
C13. Patient’s illness using up another person’s holiday/leisure time			
C14. Patient’s lack of attention to other members children and its effect on him			
C15. Any other leisure activity had to be abandoned due to patient’s illness			
Frequency distribution of Disruption of Family Interaction			
D. Disruption of family interaction			
D16. Any ill effect on the general atmosphere in the house			
D17. Do other members gets into argument			
D18. Have relatives and neighbours stopped visiting the family			
D19. Has the patient’s illness had any effect on relationship			
D20. Has the family become secluded			
Effect on Physical Health of others			
E. Effects on physical health of others			
E21. Have any other members suffered physical ill health			
E22. Has there been any other adverse effects on health			
F. Effects on mental health of others			
F23. Have any other family members sought help for pshychological illness			
F24. Has any other family members lost sleep, depressed, irritable			
